# Side branch preservation using tip detection-antegrade dissection re-entry after failed subintimal tracking and re-entry in chronic total occlusion: a case report

**DOI:** 10.1093/ehjcr/ytae571

**Published:** 2024-10-22

**Authors:** Bambang Dwiputra, Yutaka Tadano, Takuro Sugie, Tsutomu Fujita

**Affiliations:** Department of Cardiovascular Medicine, Sapporo Cardiovascular Clinic, Sapporo Heart Center, North 49, East 16, 8-1, Higashi Ward, 007-0849 Sapporo, Japan; Department of Cardiology and Vascular Medicine, University of Indonesia—National Cardiovascular Center Harapan Kita, Jl S Parman Kav 87, Jakarta Barat, 11420 DKI Jakarta, Indonesia; Department of Cardiovascular Medicine, Sapporo Cardiovascular Clinic, Sapporo Heart Center, North 49, East 16, 8-1, Higashi Ward, 007-0849 Sapporo, Japan; Department of Cardiovascular Medicine, Sapporo Cardiovascular Clinic, Sapporo Heart Center, North 49, East 16, 8-1, Higashi Ward, 007-0849 Sapporo, Japan; Department of Cardiovascular Medicine, Sapporo Cardiovascular Clinic, Sapporo Heart Center, North 49, East 16, 8-1, Higashi Ward, 007-0849 Sapporo, Japan

**Keywords:** Chronic total occlusion, Coronary intervention, Antegrade dissection re-entry, IVUS-based 3D wiring, Tip detection method, Case report

## Abstract

**Background:**

Techniques for treating difficult chronic total occlusions (CTOs) have evolved with the discovery of the tip detection-antegrade dissection re-entry (TDADR) guided by intravascular ultrasound (IVUS). This case demonstrates TDADR as a viable bailout in failed subintimal tracking and re-entry (STAR) technique.

**Case summary:**

A 78-year-old man with stable angina on optimal medical therapy had exertional angina pectoris secondary to a residual CTO lesion of the left circumflex coronary (LCX) artery. Percutaneous coronary intervention was performed for a mid-LCX CTO with a blunt proximal stump where the dissection plane expanded along the main vessel and side branch 2. Due to lack of promising collaterals for the retrograde approach, STAR successfully recanalized side branch 1. As main vessel failed to be recanalized, we proceeded with an AnteOwl IVUS-guided TDADR, intending guidewire penetration into the true lumen from the middle of the dissection plane at the main vessel, proximal to side branch 2 origin. Accurate wiring was achieved, and a guidewire was placed on side branch 2 for protection. After stent placement in the main vessel and kissing inflation, cutting balloon dilatation was performed to create re-entries for the STAR-induced extended main vessel haematoma. The procedure resulted in complete revascularization of main vessel and side branches. At 12-month follow-up, no further angina was reported, and coronary computed tomography showed patent side branches with no significant in-stent restenosis.

**Discussion:**

Imaging-based TDADR method was effective in our present case despite failed STAR technique. Limited IVUS and operator availability may become a barrier in implementing TDADR.

**Learning points**

- The tip detection-antegrade dissection re-entry (TDADR) technique plays an important role in preserving side branches during chronic total occlusion (CTO) percutaneous coronary intervention (PCI) under poor retrograde options.
- In cases where subintimal tracking and re-entry approaches encounter challenges or complications during CTO PCI, the TDADR technique serves as a viable and safe bailout option.

## Introduction

Chronic total occlusions (CTOs) pose a unique challenge in the realm of coronary intervention, demanding innovative strategies to achieve successful revascularization.^1^ Subintimal tracking and re-entry (STAR) has emerged as a valuable technique, demonstrating efficacy in navigating complex CTOs. Subintimal tracking and re-entry has been utilized as a bailout strategy, involving an uncontrolled dissection followed by recanalization into the distal lumen to restore vessel patency. However, despite its success in many cases, there are instances where STAR encounters limitations, leading to suboptimal outcomes, particularly in preserving side branches critical for maintaining myocardial perfusion.^2^ This case report explores a novel approach to address the challenges encountered following failed STAR technique in CTO percutaneous coronary intervention (PCI) by introducing the innovative concept of tip detection-antegrade dissection re-entry (TDADR).

## Summary figure

Tip detection-antegrade dissection re-entry after failed subintimal tracking and re-entry in chronic total occlusion.

**Figure:**
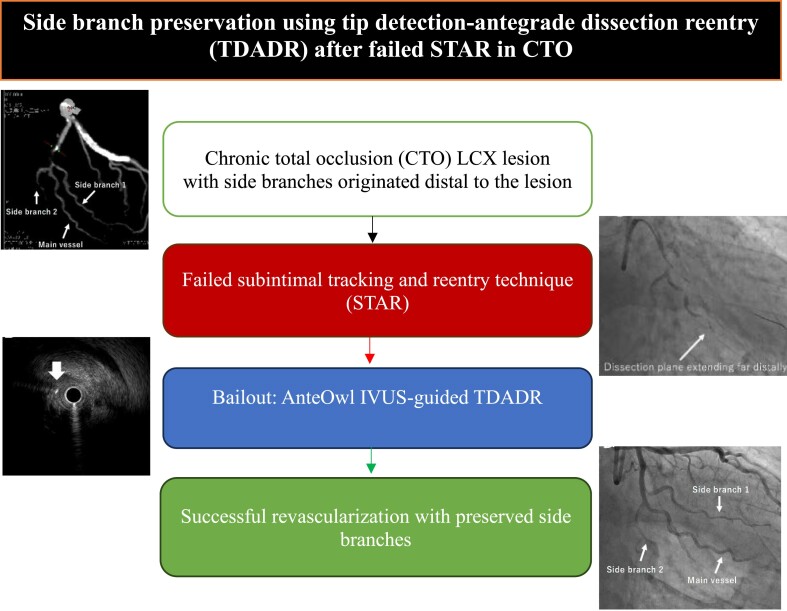


## Case presentation

A 78-year-old man with stable angina had been receiving outpatient care with optimal medical therapy. He experienced exertional angina pectoris due to a residual CTO lesion in the left circumflex coronary (LCX) artery. The patient had previously undergone successful PCI for the right coronary artery (3.5 × 20 mm stent) and left anterior descending artery (3.0 × 48 mm stent) without complications, 3 months and 3 years prior to this admission, respectively. His medical history included well-controlled hypertension and dyslipidaemia, with normal blood glucose levels, renal function, and a preserved ejection fraction. A physical examination revealed no abnormalities. His ongoing medications included aspirin 100 mg once daily, nifedipine 40 mg once daily, bisoprolol 5 mg once daily, atorvastatin 40 mg once daily, and isosorbide mononitrate 40 mg twice daily.

**Figure 1:**
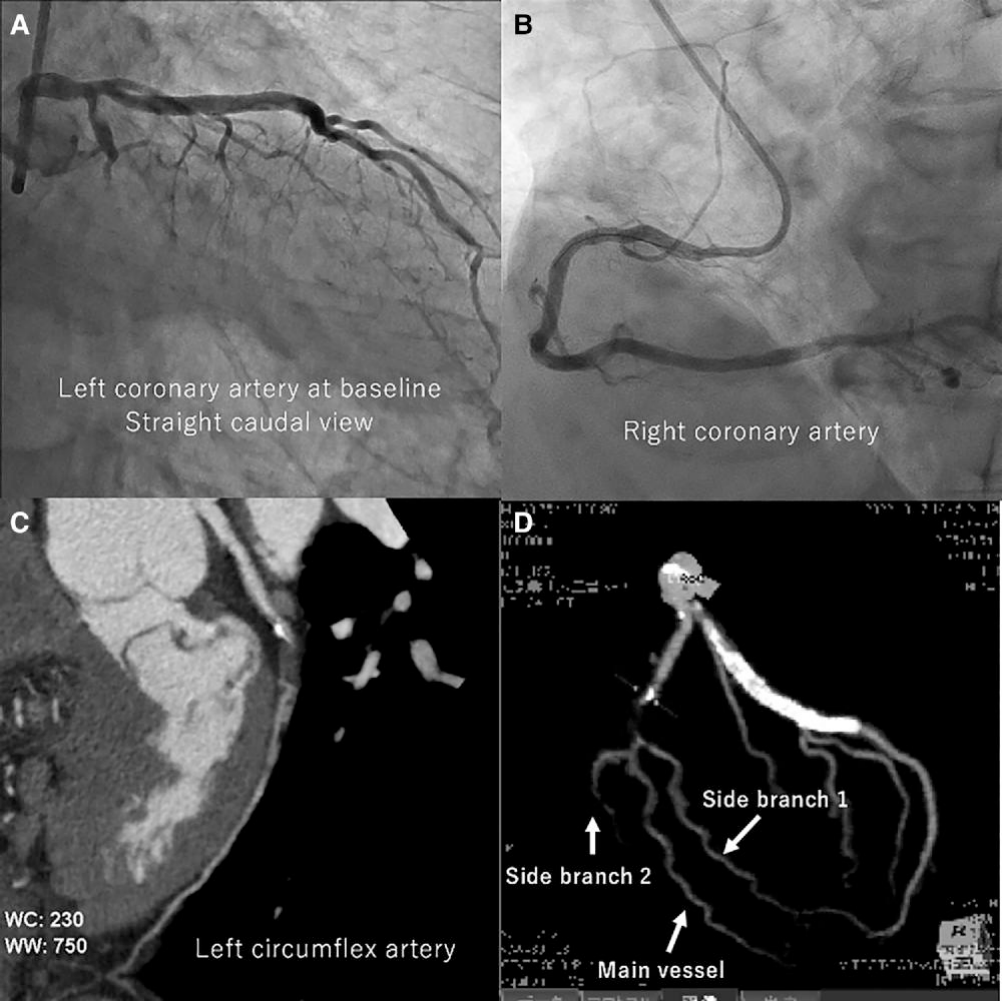
Baseline images. *(A)* The target is a chronic total occlusion in the left circumflex artery. *(B)* The right coronary artery has already been treated. *(C)* The occluded lesion has calcium at proximal cap. *(D)* The left circumflex artery has two side branches (i.e. side branches 1 and 2), which originate at sites distal to the occluded lesion.

Percutaneous coronary intervention was performed for a mid-LCX CTO with a blunt proximal stump (*Figure 1A*, Supplementary material online, *Video S1*) after treatment of the right coronary artery (*Figure 1B*). The lesion had calcium at the proximal cap (*Figure 1C*), and two side branches originating at a site distal to the CTO lesion (*Figure 1D*).

**Figure 2:**
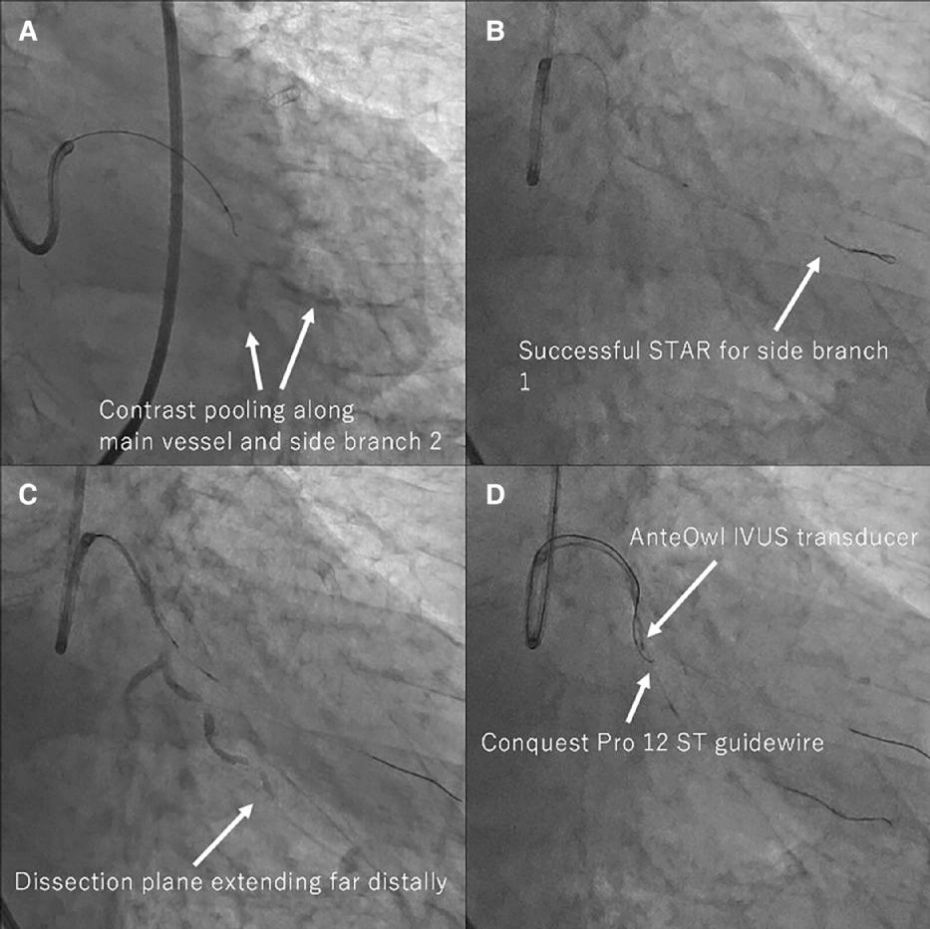
Images of the early-stage procedure. *(A)* The pooling of contrast implicates an extended dissection plane. *(B)* Side branch 1 is recanalized using the subintimal tracking and re-entry technique (STAR). *(C)* However, STAR failed for the main vessel, extending the dissection plane far distally. *(D)* We delivered AnteOwl (TERUMO, Japan) intravascular ultrasound into the dissection plane.

Using a 7 Fr AL1.5 guide, a SION black guidewire (ASAHI INTECC, Japan) first entered the dissection plane. Subsequently, the dissection plane expanded with pooling of contrast medium along both the main vessel and side branch 2 (*Figure 2A*). Subsequent guidewires were advanced only into the dissection plane. Because of the considerable time consumed and the lack of promising collaterals for the retrograde approach, we decided to perform STAR. We were able to recanalize side branch 1 using STAR (*Figure 2B*); however, we failed to recanalize the main vessel using both wire-based and contrast-guided STAR, extending the dissection plane far distally (*Figure 2C*, Supplementary material online, *Video S2*).

**Figure 3:**
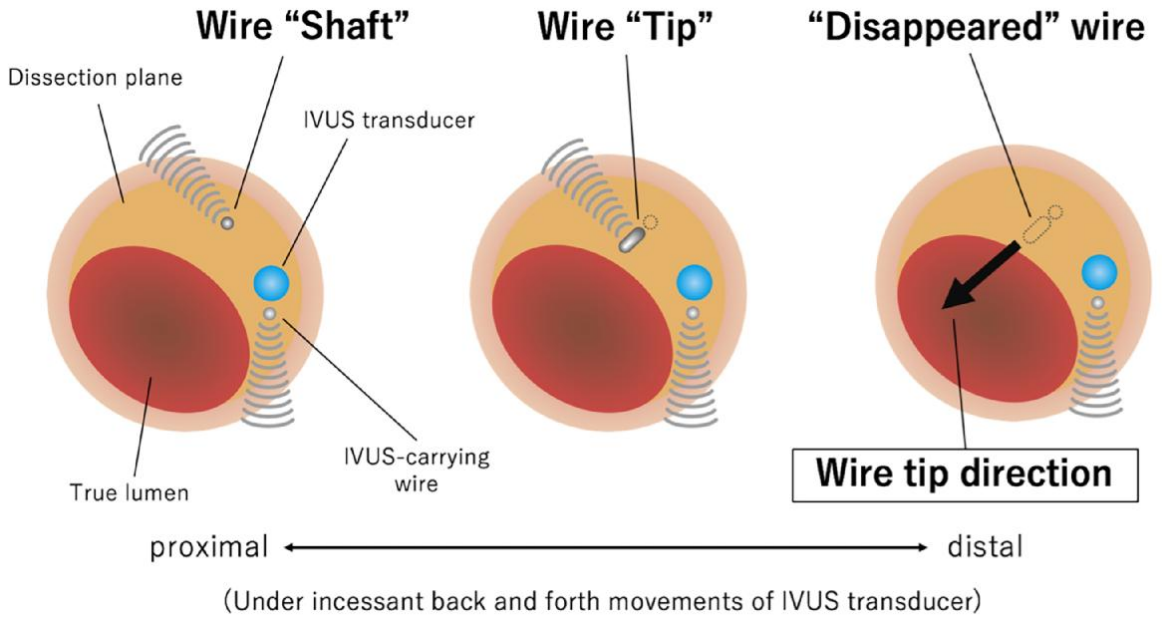
Schematic diagram describing the tip detection method of intravascular ultrasound (IVUS)-guided wiring. The tip detection method allows the visualization of the real-time guidewire tip direction with intermittent back-and-forth movement of an AnteOwl (TERUMO, Japan) IVUS transducer. While detecting the tip direction of a guidewire, we puncture the true lumen.

**Figure 4:**
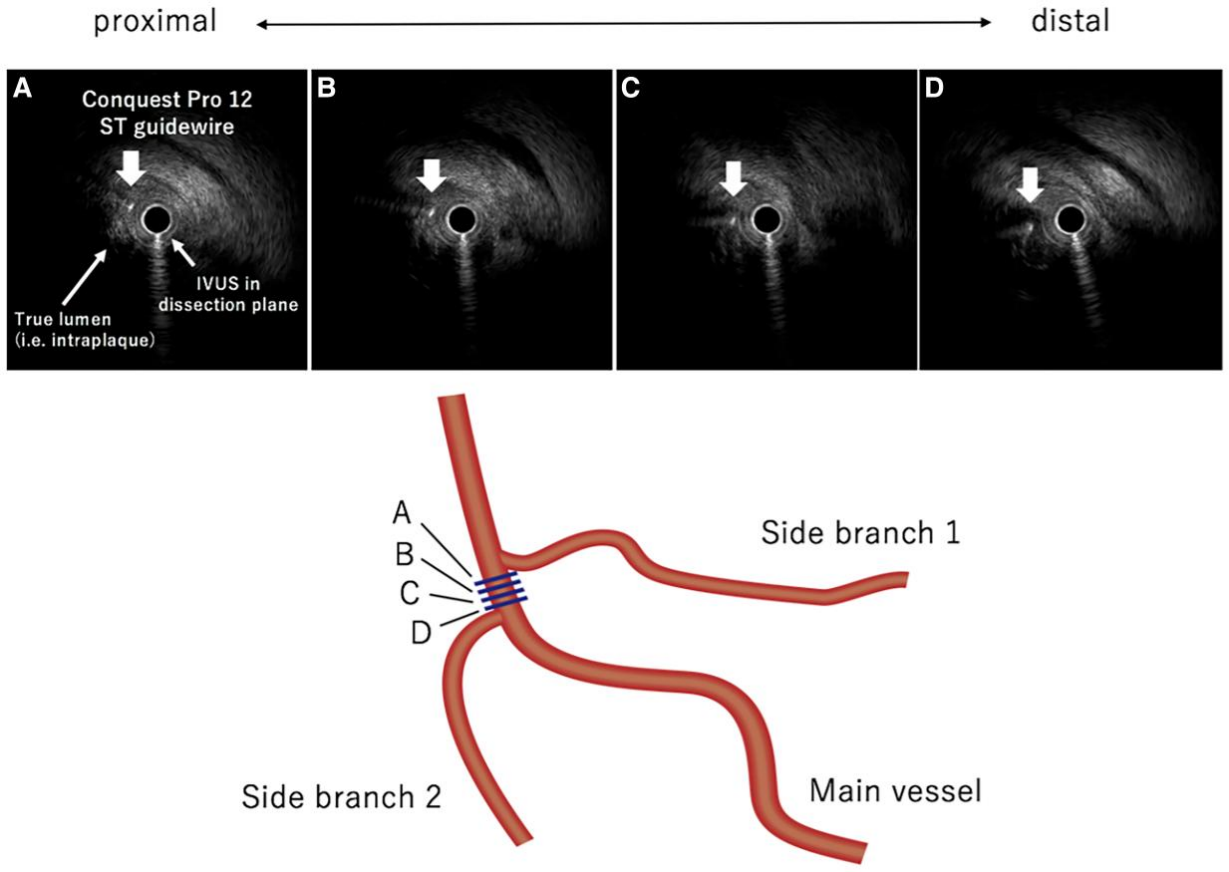
Images during imaging-based antegrade dissection and re-entry. *(A)* The vessel was positioned in the 8 o’clock direction. *(B–D)* We directed the Conquest Pro 12ST guidewire (ASAHI INTECC, Japan) tip towards the 8 o’clock position and re-entered the true lumen.

We proceeded with an AnteOwl (TERUMO, Japan) intravascular ultrasound (IVUS)-guided ADR using the tip detection method intending guidewire penetration into the true lumen from the middle of the dissection plane at the main vessel, proximal to the origin of the side branch 2. The AnteOwl IVUS was delivered into the dissection plane (*Figure 2D*). On the IVUS image, the true lumen was in the 8 o’clock direction. While detecting the direction of the Conquest Pro 12ST guidewire (ASAHI INTECC, Japan) tip with intermittent back-and-forth movements of the AnteOwl IVUS transducer, we directed its tip towards the 8 o’clock direction (*Figure 3*), punctured the true lumen wall (*Figure 4A–D*, Supplementary material online, *Video S3*) and successfully passed through to the distal true lumen.

**Figure 5:**
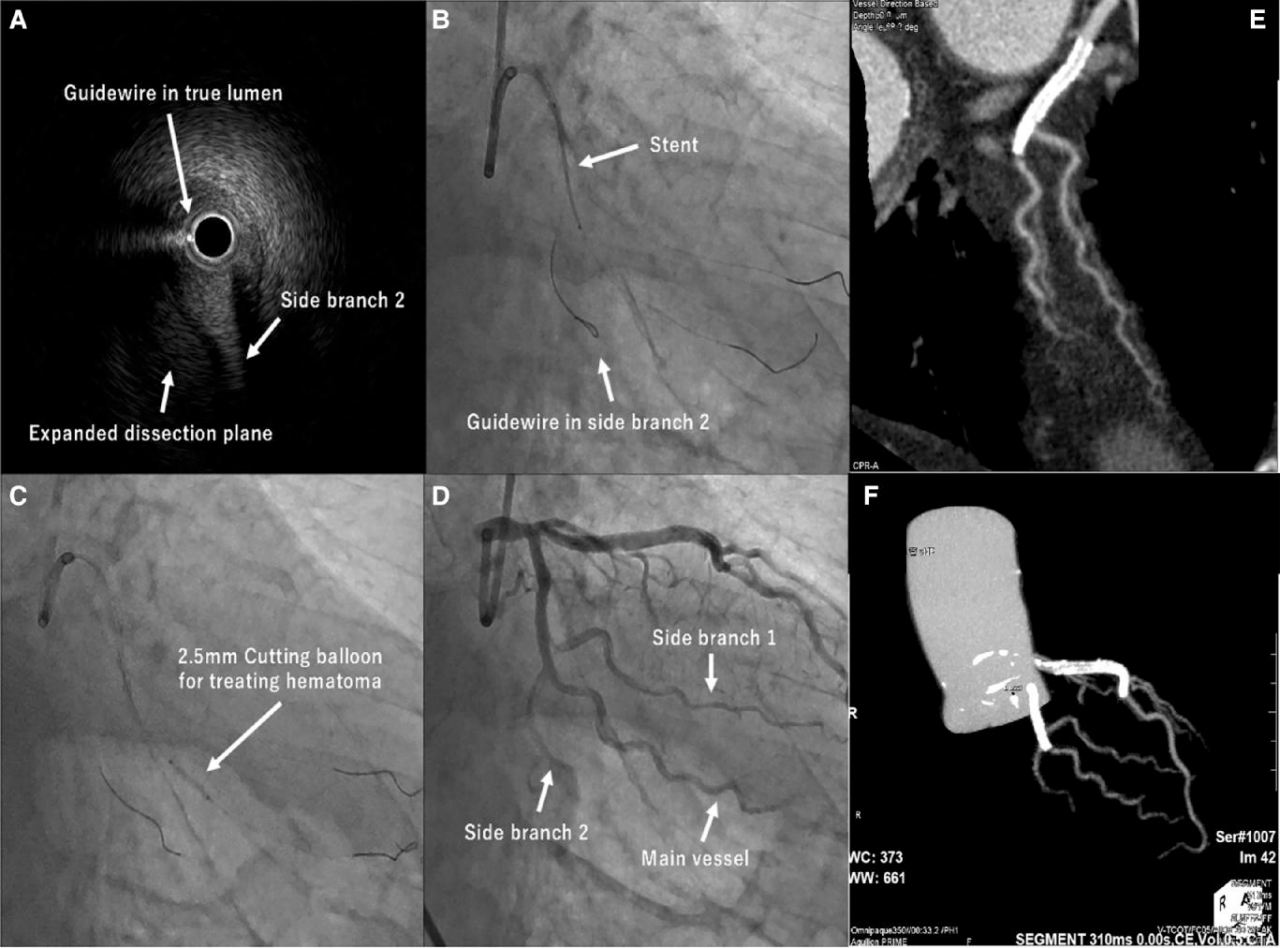
Images after the successful imaging-based antegrade dissection and re-entry. *(A)* The guidewire course was intraplaque and the side branch 2 originated from the same lumen. *(B)* After inserting a protection guidewire into side branch 2, we placed a 2.5/28 mm stent. *(C)* Cutting balloon was used to create re-entries for the huge distal site haematoma created by STAR. *(D)* Final angiography. *(E* and *F)* Follow-up coronary CT.

On the IVUS image, the distal wire course from the re-entry point was intraplaque (*Figure 5A*, Supplementary material online, *Video S4*). A guidewire was placed on side branch 2 for protection. The 2.5 × 28 mm stent was deployed in the main vessel across CTO lesion with the distal edge located within 10 mm distal to bifurcations (*Figure 5B*). After kissing balloon inflation in bifurcations, the fenestration procedure using cutting balloon was performed to decompress the extended main vessel haematoma, which obstructed distal coronary flow, caused by the STAR (*Figure 5C*). The procedure was completed with satisfactory angiographic results (*Figure 5D*, Supplementary material online, *Video S5*). The patient was reviewed at 12 months and was found to have no further angina. Follow-up coronary computed tomography (CT) showed patent side branches with no significant in-stent restenosis (*Figure 5E* and *F*).

## Discussion

We found that imaging-based ADR using the tip detection method with a Conquest Pro 12ST guidewire (ASAHI INTECC, Japan) was effective despite the presence of an expanded dissection plane after a failed STAR, thus enabling the preservation of a side branch. Preservation of side branch during PCI of a CTO lesion is essential since its occlusion has been directly associated with periprocedural myocardial infarction.^3^ In longer term, side branch occlusion can also lead to stent thrombosis, higher major adverse cardiovascular events, and cardiac mortality.^4^

Successful bailout with imaging-based ADR using the tip detection method after a failed device-based ADR^5^ and after failed wiring for a ST-segment elevation myocardial infarction patient has been reported;^6^ however, to the best of our knowledge, a successful bailout after a failed STAR has not been reported. Subintimal tracking and re-entry approach uses looped guidewire through dissection plane to distal lumen which exposes the patient to the risk of side branch occlusion during extension of dissection plane without an accurate guiding image to image bifurcation site.^7^

AnteOwl (TERUMO, Japan) IVUS-based wiring with the tip detection method allows the operator to observe the real-time positional relationship between the guidewire tip direction and the true lumen with clear image and helps the operator to determine the ideal point for vertical puncture into the true lumen when compared to Stingray ADR that were guided by unclear angiographic image.^8^ AnteOwl IVUS-based wiring also had significantly faster guidance of the CTO guidewire than the conventional IVUS-guided wiring method.^2^ Suzuki *et al*. reported the average wiring time to pass through CTO lesions was 7.3 ± 4.5 min in 15 CTO cases of IVUS-guided wiring using the tip detection method.^9^ AnteOwl IVUS also significantly enhances the success rate of IVUS-guided wiring, reduces the radiation and contrast used when compared to Navi-IVUS.^10^ Suzuki *et al.*^10^ described that IVUS-guided wiring may not be successful in true bifurcation lesions because risk of haematoma related side branch occlusion.

The tip detection method using IVUSs without an ‘in-sheath transducer pullback system’ may have a risk of broadening the dissection plane by their back-and-forth movement within dissection plane. Some mechanically-rotating transducer type IVUSs have an ‘in-sheath transducer pullback system’, however, their tip-to-transducer distance tends to be long (e.g. 20 mm in Opticross HD™: Boston Scientific, USA). Specifically developed for CTO intervention, AnteOwl IVUS has a shortened tip-to-transducer length (8 mm) and smaller shaft diameter 3.1 F.^2^ Imaging-based wiring using the tip detection method without AnteOwl was therefore difficult because of the risk of distal extension of the dissection plane by a long IVUS tip.

A conventional IVUS-guided wiring essentially does not intend for ‘ADR’, which means puncturing the true lumen wall from the middle of the dissection plane; but for a ‘reroute’, which means searching for a proximal CTO stump again intending an all intraplaque tracking.^11,12^ In the present case, imaging-based ADR using the tip detection method was accomplished using a Conquest Pro (CP) 12ST guidewire, which has an excellent penetration force with a more sharpened tip (outer diameter of the sharpened tip:0.13 mm) than Gaia NEXT (ASAHI INTECC, Japan) guidewire series (outer diameter of its micro-cone tip:0.16 mm).^13^ This hard guidewire with a 1.3 mm-long pre-shaped tip designed to be used for the purpose of ‘penetration’ bending enables an ADR even after STAR enlarged the dissection plane. It exhibits the greatest penetration force among all CTO guidewires and high success rate.^14,15^ Various guidewires have been reported for use in TDADR, such as the GAIA Next 3 (ASAHI INTECC, Japan) wire and the CP 20 wire.^5,8^ Other guidewires with high penetration profile that may be used include the Gaia Next 4 (ASAHI INTECC, Japan), Conquest Pro 40 (ASAHI INTECC, Japan), and Hornet 14 (Boston Scientific, USA).

Finally, this is a relatively advanced technique that requires both comprehensive IVUS knowledge and device availability; therefore, it may not always be globally applicable. Several case reports and descriptive studies have been published previously. We believe that this method can be adopted for clinical use around the world as the increased international availability of IVUS with a short tip with pullback system, which was first developed in Japan with AnteOwl IVUS.

## Conclusion

In the present case, imaging-based ADR using the tip detection method was effective to preserve side branches despite the presence of an expanded dissection plane after failed STAR technique.

## Lead author biography


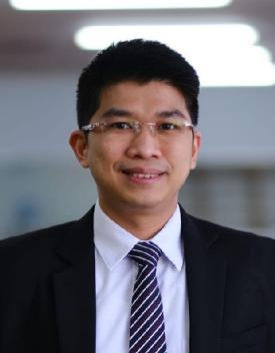
Bambang Dwiputra, MD, is currently serving as an Interventional Cardiology fellow in Sapporo Cardiovascular Center, Hokkaido, Japan. He is a cardiologist from the National Cardiovascular Center Harapan Kita, Jakarta, Indonesia and full time lecturer in Department of Cardiology and Vascular Medicine, Universitas Indonesia. Besides performing clinical works, he has contributed in international publications and residency training programme.

## Supplementary Material

ytae571_Supplementary_Data

## Data Availability

All available data are presented within the manuscript and its online Supplementary material.

## Notes

**Consent:** The authors confirm that written consent for submission and publication of this case report has been obtained from the patient in line with COPE guidance.

**Funding:** There was no funding for this manuscript.
